# Phenotypic approaches to drought in cassava: review

**DOI:** 10.3389/fphys.2013.00093

**Published:** 2013-05-10

**Authors:** Emmanuel Okogbenin, Tim L. Setter, Morag Ferguson, Rose Mutegi, Hernan Ceballos, Bunmi Olasanmi, Martin Fregene

**Affiliations:** ^1^Cassava Program/Biotechnology Program, National Root Crop Research InstituteUmudike, Abia, Nigeria; ^2^Department of Crop and Soil Science, Cornell UniversityIthaca, NY, USA; ^3^International Institute of Tropical AgricultureNairobi, Kenya; ^4^International Center for Tropical AgricultureCali, Colombia; ^5^Donald Danforth Plant Science CenterSt. Louis, MO, USA

**Keywords:** adaptation, drought tolerance, modern breeding, phenotyping, storage roots

## Abstract

Cassava is an important crop in Africa, Asia, Latin America, and the Caribbean. Cassava can be produced adequately in drought conditions making it the ideal food security crop in marginal environments. Although cassava can tolerate drought stress, it can be genetically improved to enhance productivity in such environments. Drought adaptation studies in over three decades in cassava have identified relevant mechanisms which have been explored in conventional breeding. Drought is a quantitative trait and its multigenic nature makes it very challenging to effectively manipulate and combine genes in breeding for rapid genetic gain and selection process. Cassava has a long growth cycle of 12–18 months which invariably contributes to a long breeding scheme for the crop. Modern breeding using advances in genomics and improved genotyping, is facilitating the dissection and genetic analysis of complex traits including drought tolerance, thus helping to better elucidate and understand the genetic basis of such traits. A beneficial goal of new innovative breeding strategies is to shorten the breeding cycle using minimized, efficient or fast phenotyping protocols. While high throughput genotyping have been achieved, this is rarely the case for phenotyping for drought adaptation. Some of the storage root phenotyping in cassava are often done very late in the evaluation cycle making selection process very slow. This paper highlights some modified traits suitable for early-growth phase phenotyping that may be used to reduce drought phenotyping cycle in cassava. Such modified traits can significantly complement the high throughput genotyping procedures to fast track breeding of improved drought tolerant varieties. The need for metabolite profiling, improved phenomics to take advantage of next generation sequencing technologies and high throughput phenotyping are basic steps for future direction to improve genetic gain and maximize speed for drought tolerance breeding.

## Introduction

### Importance of cassava

Cassava (manioc, yuca, or mandioca; *Manihot esculenta* Crantz, Euphorbiaceae) is an important cash crop and food crop of resource-limited farmers in Africa, Asia, and Latin America and the Caribbean. The storage roots are utilized either fresh, as in the case of sweet cultivars low in cyanogenic glycosides, or after processing into dry products such as flour, starch, and animal feed in the case of bitter cultivars high in cyanogenic glycosides (Dufour, 1988; Essers, 1995; Balagopalan, 2002; Westby, 2002). Because of its relative high productivity under conditions of erratic rainfall and low-fertility soils, 250 million Africans depend on cassava as food, with more than 90% of the 117 million tons produced in sub-Saharan Africa (SSA) in 2007 being used for fresh consumption and processed food (Philips et al., 2006). A study by the International Food Policy Research Institute (IFPRI) predicts an overall 2.44% annual growth in the use of cassava as food in SSA, closely mirroring population growth, and a growth of 1.53% per annum in cassava for feed (Scott et al., 2000).

Cassava's ability to produce in marginal environments makes it the ideal food security crop in sub-Saharan Africa where it is a staple. It can be grown with minimal inputs, but gives considerably higher yields with fertilizers and good management. The crop is flexible as to the time of harvest and can be stored naturally for long periods by keeping the plants in the field with the roots in the soil. It has a remarkable ability to tolerate and recover from biotic and abiotic stresses. Cassava offers many different alternative uses as processed food, animal feed, starch, alcohol biofuel for vehicles etc. As countries develop, their demand for all these products increases dramatically. Although pruning the aerial parts of the plant 3 weeks before harvest can reduce deterioration of the roots (van Oirschot et al., 2000), their generally short shelf-life means that they have to be used immediately or processed into dry products. Therefore, cassava processing needs to be sited near to production fields, making it an ideal vehicle for rural development through creating employment opportunities in the areas of cassava cultivation.

### Cultivated area and yield performance under optimal conditions

Cassava is widely grown in tropical and sub-tropical Africa, Asia, and Latin America, between latitudes 30°N and 30°S, from sea level to above 2000 masl on marginal and highly-eroded low-fertility acidic soils, virtually without the application of agrochemicals (El-Sharkawy, 1993, 2004; Ruppenthal et al., 1997). Africa is the largest producer of cassava with Asia as the second largest and then followed by the Americas (FAO, 2010).

Because of its metabolic efficiency under marginal conditions, cassava produces more energy per unit area than other crops under conditions of water stress and in poor soils (El-Sharkawy, 1993). By not having specific water-stress sensitive growth stages beyond storage root initiation, cassava can survive and be productive under conditions where other staple food crops, such as grain cereals and legumes, would rarely produce. Presumably, cassava originated in hot humid climates in the Amazonian lax forests (Allem, 2002). Yet it shows a high degree of tolerance to prolonged drought in areas with low and erratic precipitation of less than 600 mm annually, coupled with dry air and high temperatures (hence, high potential evapotranspiration), low fertility soils and high pest and disease pressure such as in Northeastern Brazil, the northern coast of Colombia, the coast of Peru, the Sahelian areas of West Africa, and drought-prone areas of East and Southern Africa, and parts of Thailand (El-Sharkawy, 1993). Particularly notable is cassava's recent expansion into the Sahelian parts of West Africa where its tolerance to various edaphoclimatic stresses gives an advantage over other staples (Romanoff and Lynam, 1992; El-Sharkawy, 2004).

Cassava has a high yield potential; when compared to maize, sorghum and rice in environments with no production constraints, it can match or exceed these crops in energy production per hectare (De Vries et al., 1967). Average annual cassava yields worldwide are 10 t ha^−1^ (fresh root; about 65% moisture content), ranging from 6 t ha^−1^ in Mozambique to 26 t ha^−1^in India. Yields as high as 90 t ha^−1^ have been obtained in experimental trials of some improved cassava cultivars under near-optimum climatic conditions in Colombia (El-Sharkawy, 2005).

The gap between the potential yield and actual yields in farmers' fields is around 8-fold, suggesting that the highest potential of cassava production is far from being reached with traditional varieties, usually cultivated on marginal soils without inputs. Thus, a small increase in cassava yield in these marginal regions resulting from the use of improved drought-tolerant varieties would easily lead to an increase in global production. Many cassava genotypes have been identified that are very well adapted to drought and have been released in specific regions. However, it is necessary to understand the genetic and physiological traits that lie behind the mechanisms that make cassava a renowned drought-tolerant crop with the capacity for further progress, and for the application of these principles in breeding programmes for cassava and other crops.

### Genetic and genomic resources

There are several germplasm collections with hundreds to thousands of cassava accessions in the national programmes of Brazil, Mozambique, Nigeria, Tanzania, and Thailand. In addition, two International Agricultural Research Centers, Centro Internacional de Agricultura Tropical; International Center for Tropical Agriculture (CIAT) in Colombia and International Institute of Tropical Agriculture (IITA) with its headquarters in Nigeria, hold large collections. The collection at CIAT comprises more than 6000 accessions, and has been evaluated for pest and disease resistance and for novel starch quality traits (Bonierbale et al., 1995; Bellotti, 2002; CIAT, 2002). IITA's collection consists of nearly 2000 genotypes largely of West African origin. This germplasm has been systematically characterized and evaluated for disease response. Understanding the distribution of genetic diversity within and among individuals, populations, species, and genepools is crucial for the efficient management of germplasm collections.

Molecular markers are playing an increasing role in germ-plasm characterization. Beginning in the early 1990s, molecular and genomics tools were developed to elucidate the genetics of traits of economic value in cassava. Genetic markers developed for cassava include more than 3000 restriction fragment length polymorphism (RFLP), 800 simple sequence repeat (SSR), 120 random amplified polymorphic DNA (RAPD), and nine isoenzyme markers (Fregene et al., 1997; Mba et al., 2001; Okogbenin et al., 2006; Raji et al., 2009).

A total of a non-redundant set of 2146 SSRs comprised of 1675 curated from the genome and 471 curated from ESTs are now available (Ferguson et al., 2012b). The curated, paired SSR sets and associated information can be found at http://bioinformatics.iita.org/cassava_SSRs. SSR markers have also been employed to study the genetic diversity and structure in a large collection of local varieties from Africa and Latin America, the results of which have been deposited on the website of the Generation Challenge Programme (GCP) of the Consultative Group on International Agricultural Research (CGIAR)^1^. Data from a molecular diversity assessment of over 1000 varieties from seven countries in Southern, East and Central Africa are also available at the same site.

Many molecular genetic linkage maps have also been constructed for cassava. The first was constructed using an intraspecific F_1_ cross and 612 RFLP, RAPD, SSR and isoenzyme markers (Fregene et al., 1997). The second map was constructed using 100 SSR markers and an S_1_ family (Okogbenin et al., 2006). Recently new maps have been published (Chen et al., 2010; Kunkeaw et al., 2010, 2011; Sraphet et al., 2011; Whankaew et al., 2011). Molecular markers linked to resistance to cassava mosaic disease (CMD), cassava green mite (CGM), and cassava bacterial blight (CBB), β-carotene content, and early root yield have also been identified (Akano et al., 2002; Okogbenin and Fregene, 2002).

Diversity Array Technology (DArT) can be used to characterize several hundreds to thousands of polymorphisms in a timely, cost-effective manner (Ferguson et al., 2012b). The first developed cassava DArT array had nearly 1000 polymorphic clones with a 99.8% reproducibility (Xia et al., 2005), offering a high-throughput marker screening system at a low cost.

Recently, the Generation Challenge Program (GCP) converted 1740 SNPs in cassava for use on the KASPar platform (LGC). Through the GCP IBM marker services (http://marlow.iplantcollaborative.org/marker-service). To date, 80,631 cassava ESTs have been deposited in GenBank (Lopez et al., 2004; Lokko et al., 2007; Sakurai et al., 2007; Ferguson et al., 2012a,b).

Four bacterial artificial chromosome (BAC) libraries have been generated in cassava for positional cloning and genome sequencing. The first library is from the CMD-resistant genotype TMS 3001 and it has a 5X genome coverage. The second library is from the whitefly-resistant genotype MECU72 with a 10X genome coverage, while the third was constructed from TME3, a CMD-resistant genotype, with an 11X genome coverage. The fourth library was constructed from AM560-2, a partially inbred genotype for physical mapping and sequencing of the cassava genome. These BAC resources are available from the Clemson University Genomics Institute (CUGI^2^ ). More than 2000 BAC ends have been sequenced in cassava and another 5000 are currently being sequenced.

A 22X genome sequence of cassava was recently completed via shotgun and a 454 Genome Sequencer FLX platform with long-read GS FLX Titanium chemistry. More than 61 million sequencing reads were generated and assembled into a draft genome that contains an estimated 95% of cassava genes. It is one of the first large genome projects to primarily use the 454 Life Sciences^3^ long-read sequencing platform, which enabled both improved quality of the draft and its rapid generation. The annotated draft genome sequence is available at the United States Department of Energy Joint Genome Institute (DOE–JGI) Phytozome website^4^.

### Improving cassava adaptation to drought

Cassava's huge potential to produce well in marginal environment has made it a desirable and strategic crop for increasing food productivity by exploring the vast arable lands in the semi-arid and arid ecologies in the tropics. The wealth of genetic resources and the genetic diversity it offers has been deployed in the genetic improvement of cassava for drought tolerance. This has resulted in the identification of useful mechanisms associated with cassava's adaptation to drought. The mechanisms essentially combine dehydration avoidance, dehydration tolerance and those linked to optimum growth and metabolism. Several traits have been identified associated to these mechanism. Genetic improvement for drought adaptation in cassava is being enhanced by the increasing volume of genomic resources that are now available to breeding programmes. The ability to take maximum advantage of these genomic resources in modern breeding platforms essentially depends on improving capacity for drought phenotyping. Among the attributes for desirable drought phenotying traits in modern breeding are that it must be genetically associated with yield under stress, highly heritable, genetically variable, cheap, and fast to measure, stable within measurement period, and must not be associated with yield penalty under unstressed conditions. Modern breeding essentially entails combining genetic and genomic resources with good phenotyping protocols to efficiently breed for drought adapted varieties with speed and genetic again. This paper reviews drought adaptation studies in cassava, the critical phenotyping needs for modern breeding of drought tolerance and the future direction in terms of simple but advanced phenotyping methodologies that can further elucidate and enhance a better understanding of the mechanisms underlying drought adaptation in cassava.

## Phenotyping for drought tolerance in cassava

### Field experimentation requirements

The key requirement for field trials to measure drought tolerance is to have appropriate water stress conditions. Achieving proper control over the field stress environment in order to assure the relevant drought test profile can be problematic. The common test criterion is yield when yield under stress is the target of the breeding programme. Yield under stress may be affected by the genetic makeup of yield potential and by specific genes affecting drought resistance. Estimating drought resistance in terms of the yield difference between potential and stressed growing conditions can isolate the two effects.

Well-designed and planned field experiments to determine relevant physiological traits are the most cost-effective way of evaluating drought tolerance in cassava. Ideal sites are semi-arid regions with more than 600 mm of rainfall distributed over 3 months of the year. Cassava requires 3 months of rainfall or irrigation for establishment of the crop, after which, tolerance to drought can be measured effectively. Sites in Colombia used by CIAT for the evaluation of drought tolerance include:

- Rio Hacha, Guajira Department: latitude: 11°32′40″N; longitude: 72°54′26″W.
- La Tatacoa desert (Villavieja), Huila Department: latitude: 3°13′22″N; longitude 75°13′21″W.

In Africa, IITA and national programmes use sites at:

- Hombolo, Tanzania.
- Kiboko and Kibwezi in Kenya.
- Minjibir (Kano State) in Nigeria.

The selected field should be flat, of well-drained and more or less homogenous soil. A simple randomized complete block design (RCBD) of 20 plant plots (4 × 5) and six replications can be used when planting material is abundant and not a limiting factor. Otherwise, single-row plots of six to eight plants can be used. It is preferable to have a higher number of replications and smaller plot sizes to reduce environmental effects. Where more than 200 genotypes are to be evaluated, it is preferable to divide the experiment into two or more individual experiments to limit the size of the field, and thereby limit environmental variation. Two treatments should be imposed: well-watered and water-stressed. The response to water stress of a genotype can then largely be assessed through differences in yield under the two treatments. In certain environments, a genotype with average yield potential, but little yield penalty under drought stress may be preferable to a genotype with high yield potential but a large yield penalty under drought stress. When response to late stage, long duration drought is being assessed, irrigation should be applied to both treatments for the first 3 months to get even plant establishment. Irrigation can then be withheld on one treatment 3 months after planting. If early drought is to be assessed irrigation should be withheld 2–3 weeks after planting.

Measurement of physiological traits normally begins at 3 months, at the onset of water stress and continue every month until harvest. Harvest related traits, for example harvest index (HI) and fresh root and foliage weight, are typically measured at 12 months after planting. Stomatal conductance and photosynthesis should be measured using field-portable gas exchange equipment, and the leaf canopy measured with a leaf canopy meter. Soil measurement is also very important to monitor stress levels. Soil cores are taken periodically at 0.3 m intervals within eight profiles from stressed and unstressed plots. The soil samples are immediately weighed and then oven dried at 70°C to a constant weight. Volumetric soil water content is often determined taking into consideration the soil bulk density at each layer. Simple equipment like the soil tensiometer or more sophisticated equipment such as the neutron probe can also be used to measure soil moisture content.

### Conventional traits measured in drought adaptation studies of cassava

Pre-harvest traits are normally measured on a monthly basis beginning at three MAP and they include:

1. Number of primary stems.
2. Number of branching levels.
3. Length of primary and secondary stems.
4. Leaf retention using two methods: (1) percentage method (visual score), and (2) leaf scar method, a more quantitative measure of leaf retention.
5. Height of leafless stem.
6. Length and width of fully expanded leaf lobe.
7. Carbohydrate content of leaves, stems, and petiole.
8. Stomatal conductance and ABA content of leaves and stems.
9. Pest and disease incidence.

The traits measures at harvest at 12 MAP are:

1. Above-ground biomass.
2. Storage root fresh weight.
3. Number of storage roots.
4. Stem diameter.
5. Storage root dry matter (percentage).
6. Storage root starch content (percentage).

### Drought tolerance response in cassava

The physiological responses of cassava to water stress and possible mechanisms underlying the crop's tolerance to drought have been the subject of several studies (Connor and Cock, 1981; Connor and Palta, 1981; Cock, 1985; El-Sharkawy and Cock, 1987; Alves and Setter, 2000, 2004a; Ekanayake and Ginthinguri, 2000; Okogbenin et al., 2003; El-Sharkawy, 2004; Lenis et al., 2006). Various methodologies and mechanism have been reported for drought stress tolerance studies in cassava.

#### Stomatal conductance

Measurement of the stomatal control of water loss can be valuable in identifying desirable genotypes. In studies conducted on 10 cassava clones evaluated for drought tolerance at the IITA Minjibir station, the stomatal conductance of the lower leaf surface was measured using a porometer (Mk3, Delta-T Devices, Cambridge, England) and CO_2_ fixation was measured with a leaf disc electrode (LD2, Hansatech, Norfolk, England). These studies showed that, while most clones increased stomatal conductance throughout the day, at 4 months after the last rains, clones TMS91934 and TMS84751 diminished their stomatal conductance after 14:00 h and 12:00 h, respectively (CIAT report, 1994). Apparently, these clones gave a better yield because they depleted the soil water more slowly. In such cases, optimizing crop water-use efficiency (WUE) is of greater importance than maximizing short-term growth—until the soil water is depleted, at which point growth is halted (El-Sharkawy, 1993).

#### Leaf formation and other growth parameters

The growth of cassava has been evaluated to assess the effect of drought stress on the crop's physiology with particular emphasis on leaf formation and other parameters such as plant height and number of active apices. In earlier work at IITA, Kano (Okogbenin et al., 1998), the proximity of an artificial lake to experimental station was used to set up different stress sites reflecting different water table depths. Five experimental sites representing water table depths over two seasons were used in drought tolerance studies at IITA's research station at Minjibir in Kano state in the Sudan savannah ecology. Experimental sites within 100 m of the lake were arbitrarily assigned to the high water table (HWT) section, while sites between 100 and 200 m from the lake were assigned to the intermediate water table (IWT) section. Field sites further than 200 m from the lake were assigned to the low water table (LWT) section. Nine varieties were evaluated in the first season, while eight clones were evaluated in the second season. The locations chosen for this study were sufficiently diverse to produce observable differential varietal responses to seasonal and site-specific moisture gradients at Minjibir. Data on different water table depths at various locations at Minjibir were available from previous studies (Ekanayake et al., 1996; Okogbenin et al., 1998, 1999) using the same sites. The varietal response differed with water table section, indicating differential adaptation responses to drought stress among varieties.

In the drought-stress study conducted at Minjibir, Nigeria (Okogbenin et al., 1998), differences in soil water content were established by planting two identical trials at different distances from an artificial lake, assuming that there were differences in the water table at the two sites. Soil water measurements taken using a neutron probe in 60 access tubes installed in all experimental plots showing significant difference in relative water contents during the dry season as a result of the differences in water table caused by the proximity of the lake. This was especially so in the deeper layers of the soil profile until 22 weeks after the last rains. This illustrates an approach that could be used in the absence of irrigation facilities.

In this drought study at IITA, the number of newly formed leaves on an apex, the number of nodes, the number of active apices and the number of fallen leaves on one apex were measured at regular intervals, depending on the particular experiment. The number of fallen leaves was obtained by counting the number of bare nodes on one apex. Plant height was measured from the soil surface to the general height of the canopy.

Results suggest a strong influence of the water table on growth (as reflected in height and leaf formation) and leaf shedding of the 10 clones under examination. Plant growth represented by plant height measured at 3-week intervals starting ca 20 weeks after the rains was higher at the IWT sites when compared with data obtained from plants growing away from the lake (LWT sites); the differences increased with time after the rains. A similar effect of the depth of the water table was observed in the cumulative number of leaves formed per plant. Using the initial number of leaves per plant at the beginning of the dry season as a covariate, it was possible to detect statistically significant differences after 6–9 weeks. Plants growing closer to the lake were able to form more leaves than those at LWT sites.

The leaf area index (LAI) refers to the leaf area per unit area of ground. The maximum LAI in cassava ranges from 4 to 8, depending on the cultivar and the atmospheric and edaphic conditions prevailing during crop growth (Cock, 1985). At the onset of the dry period, the cassava crop reduces its leaf area by producing fewer and smaller leaves, and by shedding older leaves. The reduced leaf area in dry weather could be considered a means by which cassava reduces water loss by transpiration. However, reduction in leaf area during long periods of water stress also reduces the crop growth rate. The reduction is more pronounced in the shoots than in the roots, particularly in varieties with vigorous vegetative growth. Upon recovery from water stress, cassava rapidly regenerates new leaves and the LAI of previously stressed cassava plants becomes higher than in non-stressed plants (El-Sharkawy and Cock, 1987).

Dry periods also cause a decline in LAI. After a decline during the dry season, and followed by a second rainy season, leaf area may increase a second time, but may not be as high as in the first season (Osiru et al., 1995). Selection for higher top weight and hence higher LAI should favor high root yield, since there is an optimum relationship between root yield and LAI. Drought resistance determines the base yield under stress, while recovery prescribes the upper potential after stress. Rapid production of new leaves in the recovery phase with the commencement of rainfall toward the end of the growth cycle (Ekanayake, 1993) could stimulate greater accumulation of assimilates in the roots of highly vigorous varieties such as TMS90257, TMS91934, TMS50207, and TMS30572 after the dry season and just before harvest.

Leaf area density (LAD) is the integral of LAI over time. LAD is calculated by multiplying LAI with the time (in days or weeks) during which the leaf area is photosynthetically functional. Good examples of long-LAD varieties developed at IITA are TMS91934 and TMS4(2)1425 (Osiru et al., 1995).

#### Water-use efficiency

WUE has been used to evaluate drought tolerance in cassava. Regarding the water extraction capacity of the different clones in situations of water availability in the IITA study described above, those with higher yields in the LWT site showed a tendency to extract less water from the deeper layers of the soil (120–180 cm) during the first 24 weeks of measurements. TMS90853 extracted 64%, 51%, and 49% of the available moisture at 10, 150, and 180 cm, respectively in the soil profile. TMS50207 extracted 60%, 57%, and 35% at the same respective depths. However, under water stress, TMS90853 had higher top growth than TMS50207, which explains the high amount of water extracted at deeper levels by TMS90853. The clones that extracted higher percentages of the water from deeper soil layers (TMS4(2)1425 and TMS84751) had the lowest root yield, showing poor WUE. However, TMS91934 and TMS84751 formed too many leaves in at the LWT site and also shed a high number of leaves at the same location, suggesting inefficient utilization of the little water available in the soil profile. Under stress, TMS30572 showed a reduced yield of 39% and 44% of shoot and root weight, respectively (Okogbenin et al., 2003).

#### Leaf scars and leaf life

Cassava adapts to water shortage by reducing its leaf canopy (Connor et al., 1981; El-Sharkawy and Cock, 1987) to reduce water use. Hence, leaf shedding is an effective adaptation mechanism as a response to moisture stress. In the drought experiment at IITA, one of the youngest leaves (not unfolded length approximately 1 cm) per plant of all sample plants was regularly (generally once a month) tagged with a label on which the clone, plant number, date of labeling, and replication number were coded. The tagged leaves that had fallen were collected every week, enabling the life of individual leaves formed at different plant ages to be calculated.

Leaves dropped as a percentage of leaves formed increased from HWT to LWT sites. Thus, it may be desirable to breed and select for better leaf retention when developing varieties adapted to dry areas. The reduction in leaf canopy could not be attributed solely to observed leaf fall since, at the IWT (moderately stressed) site, more leaves were shed than at the LWT site. Plants at sites supporting vigorous growth were more likely to develop a very dense leaf canopy. Mutual shading of leaves limits leaf life and accelerates leaf senescence in such plants (Rosas et al., 1976). This may be responsible in part for the high rate of leaf shed at IWT compared with LWT sites. However, genetic variation for leaf scars was minimal amongst varieties. Because vigorous clones were more likely to shed more leaves and vice versa, the number of leaf scars was, as expected, strongly associated with the number of leaves formed.

#### Leaf photosynthesis

The use of leaf photosynthesis as a selection criterion in cassava improvement programmes might be difficult to handle when evaluating large breeding populations. Despite this, canopy-scale photosynthesis, which can be evaluated by measuring crop biomass growth rate, should be included at least in the evaluation and selection of parental materials. Its use should be combined with other important yield-related traits, particularly a relatively high (>0.5) harvest index (HI; Kawano, 1990, 2003, a large root sink using root number per plant as an indicator (Cock et al., 1979), and longer leaf life, i.e., greater leaf retention and duration over the growth cycle (El-Sharkawy, 2004; Lenis et al., 2006). Recent advances in molecular biology and the development of more precise techniques and equipment will further enhance and accelerate the elucidation of the fundamental mechanisms underlying photosynthetic potential and associated beneficial traits and their controlling genes.

Breeding at CIAT, while diversifying the genetic base, has incorporated many such accessions for their useful plant traits. Outstanding among the accessions used is the clone MBRA12. This exhibits high leaf photosynthesis when grown outdoors in pots or in the field in a mid-altitude warm climate and high yield coupled with resistance to mites (Byrne et al., 1982). Other accessions of Brazilian origin, MBRA383 and MBRA191 that ranked highly in this group of clones, were also reported to be among the highest ranked clones (fourth and fifth, respectively, among 33 evaluated) (El-Sharkawy, 2004).

#### Storage root and shoot harvest

In the IITA experiments described earlier, the internal samples were collected at harvest. The plants were separated into leaves, stems, original stem cuttings and storage roots, and bulked per plot. The root fresh weight was measured and the HI was calculated by expressing the root yield as a fraction of the total biomass.

At harvest (12 MAP), the root yield of certain varieties at the severely stressed site (LWT) approached that of the less stressed sites (IWT and HWT). El-Sharkawy (1993) reported that leaves of plants growing in highly stressed environment tend to have higher stomatal conductance than leaves of similar ages in unstressed plants. Varieties TMS63397, TMS50395, TMS84751, and TMS4(2)1425 did not show much differences in final root yield among the different water table sections (Okogbenin et al., 2003).

Varieties with a good top weight tended to produce a good top yield. Previously work (Connor et al., 1981) suggests that vigorous genotypes produce better under stress than less vigorous types. Therefore, a variety with optimal leafiness under good conditions is required for the attainment of a high yield in both high- and low-stress conditions, (Ekanayake and Ginthinguri, 2000). The relative reduction in yield caused by water stress was used to assess the relationship between drought resistance and yield performance.

**Figure 1:**
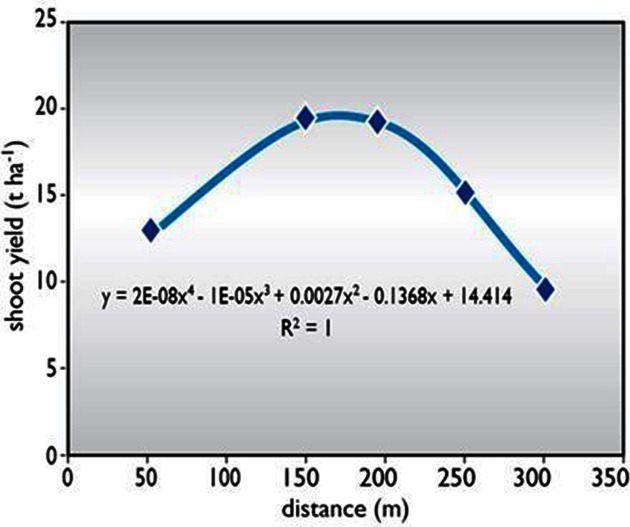
**The relationship between fresh shoot yield and water table depth as a function of field location (distance from lake) at IITA's research station at Minjibir, Kano, Nigeria.** Source: redrawn from Okogbenin et al. (2003).

**Figure 2:**
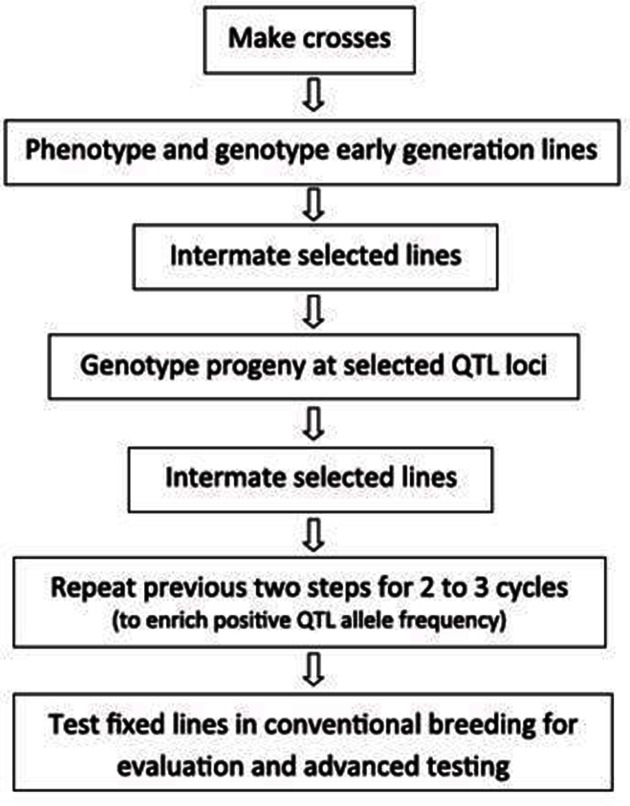
**MARS scheme (adapted from Integrated Breeding Platform—Generation Challenge Programme) (Ferguson et al., 2012b)**.

Average fresh shoot yield was higher at the IWT site than at the LWT site (Figure 1; Okogbenin et al., 2003). The reduction in crop growth was more pronounced in the shoots than in the roots, particularly in varieties with vigorous vegetative growth. Results revealed a 37% reduction in fresh root yield, compared with a 22% reduction in fresh root yield from the IWT site to the most severely stressed LWT site. A highly significant non-linear relationship was observed between fresh shoot yield and water table depth (Figure 2). Fresh shoot yield is a parameter of economic importance in dry ecological zones where animal feed supply is critical during the dry season. Therefore, varieties that produce abundant foliage are desirable as a source of feed.

In summary, conditions at the IWT site were more conducive to cassava growth than those at the HWT or LWT sites. Total plant biomass was higher at the IWT site (41 t ha^−1^) than at the LWT (29 t ha^−1^) and HWT (24 t ha^−1^) sites (Okogbenin et al., 2003). The seemingly poor growth observed at HWT may have been caused by waterlogged conditions (due to inadequate drainage) that persisted in the first 3 months of rainfall, thus interfering with initial plant growth and development at this location. Compared with results at the IWT site, decreases at the LWT site were 23% for leaves formed, 19% for leaf scars, 22% for root yield and 37% for shoot yield. Again, compared with the IWT site, decreases at the HWT site were 25% for leaves formed, 27% for leaf scars, 50% for root yield and 32% for shoot yield. The greater reductions at the HWT site are in agreement with earlier findings that cassava tolerates stress from drought better than from water-logging (Lahai et al., 1999). Severe drought stress at the LWT site caused a reduction in growth in this section of the field.

#### Fibrous root measurements

Evaluation for deep fibrous root system for drought measurement is relatively difficult in cassava. Cassava roots could be as long as 2 m in the ground and could help cassava to have access to deep water layers and may be deployed by the crop as escape mechanism from to evade water stress. Studies were conducted at Ibadan with the objective of identifying differences in the fibrous root system of 10 IITA genotypes, using early root growth as a selection criterion for adaptation to drought. This revealed large genotypic differences in fibrous root weight and root length as measured 2–5 weeks after planting (WAP) (CIAT report, 1994).

#### Water stress responses

Several reviews of cassava's tolerance to water stress have been produced (El-Sharkawy, 2004; Setter and Fregene, 2007). These reviews reveal that the principal mechanisms that may control tolerance to drought in cassava include its sensitivity and response to changes in atmospheric humidity and soil water status El-Sharkawy and Cock, 1984. Thus, once exposed to dry air and/or dry soils, cassava leaves partially close their stomata thereby restricting water loss. They are also capable of partially retaining their photosynthetic capacity under prolonged water shortage. Considerable variation has been observed in leaf conductance and this parameter seems to be useful to preselect sources of germplasm conferring adaptation to prolonged dry periods [Empresa Brasileira de Pesquisa Agropecuária, Brazilian Agricultural Research Corporation (Embrapa, 1992; Iglesias et al., 1995)].

Cassava is capable of forming deep rooting systems (below 2 m soil depth) that allow the crop to extract storage water when available (El-Sharkawy, 2004). Although cassava has sparse fine root systems compared with other crops such as cereals and tropical grasses, it is capable of penetrating into deeper soil layers.

Cassava conserves water under extended stress by reducing light interception, achieved through a reduced leaf canopy via restricted formation of new leaves, production of leaves of a smaller size, drooping of the leaves (“heliotropic response”), and leaf fall (Porto, 1983; El-Sharkawy and Cock, 1987; Calatayud et al., 2000; Alves and Setter, 2004b). Although a reduction in leaf area conserves water, it would also lead to a reduction in total biomass and yield (Connor and Cock, 1981; El-Sharkawy and Cock, 1987; Connor et al., 1981; Porto, 1983; El-Sharkawy et al., 1992; El-Sharkawy and Cadavid, 2002). However, upon rewatering, cassava can recover rapidly by forming new leaves. This increases light interception and canopy photosynthesis, thus compensating for previous losses in biomass, particularly root yield.

#### Osmoregulation under extended water stress

One means of increasing drought tolerance is by accumulation of osmotically active solutes, so that turgor and turgor-dependent processes may be maintained during episodes of dry down. In some plant species, osmotic adjustment (OA) allows cell enlargement and plant growth under high water stress, and allows stomata to remain partially open and CO_2_ assimilation to continue at low water potentials that would otherwise be inhibitory (Pugnaire et al., 1994). However, the extent of OA has been found to be quite limited in cassava's tolerance to drought. In studies with potted greenhouse-grown cassava, Alves (1998) found that the largest increases in solutes after a few days of water deficit occurred in the youngest and folded (i.e., expanding) leaves, with only about 0.14 MPa OA increase in mature leaves, pointing to a limited effect of OA in the latter leaves (Alves and Setter, 2004a). Such studies need to be carried out on field-grown plants subjected to gradual, prolonged water stress to ensure that they extrapolate to field conditions.

#### Abscisic acid accumulation

Under drought, changes in the biosynthesis, content and distribution of plant growth regulators such as abscisic acid (ABA) within plant organs and tissues—particularly in roots, leaves, and buds—may play an important role in sensing changes in both soil water and atmospheric humidity, and in controlling stomatal movements, leaf formation and extension, root growth and bud dormancy. They may also be involved in other biological functions such as the expression of dehydrin and other proteins that are thought to stabilize macromolecular structure (Alves and Setter, 2000, 2004a).

Studies by Alves and Setter (2000) showed that five cassava varieties rapidly accumulated large amounts of ABA coincident with the cessation of leaf expansion growth and transpiration. The high ABA readings were almost completely reversed to control levels after 1 day of rewatering. This rapid return to control levels corresponded with a rapid recovery of leaf area growth rates. A substantial proportion of the variation in ABA concentration in cassava correlated with genotype, suggesting that genetic variation for the trait might be found in cassava.

#### Critical period for drought tolerance

In general, for cassava grown in a range of environmental conditions, there is a positive correlation between the total biomass and storage root biomass. However, during growth, there are distinct developmental phases. During the first 3 months, cassava accumulates dry matter more in the leaves than the stems and tuberous roots. After the third month, more is accumulated in the roots than the rest of the plant (Ghosh et al., 1988).

Connor et al. (1981) reported that, when rainfall was withheld from cassava for 10 weeks commencing 12 weeks after planting, tuber yield was reduced by 32% compared to the control. Oliveira et al. (1982) imposed a water deficit for 2 months during successive 2-month periods from the first up to the 11th month after planting (MAP) and they found that a critical period for cassava root yield extended from the first to the fifth MAP. This period corresponds to the stages of root initiation and bulking. Water stress in this period reduced storage root yield by 60%. A similar conclusion was reached by Porto et al. (1989), who evaluated cassava grown in a lysimeter with water-stress conditions imposed over a 100-day period with no water, starting at three and six MAP. They reported that the accumulation of total dry matter as well as root dry weight was reduced more by water stress beginning at three than at six MAP. Thus, the more severe effect corresponded to stress during the period of rapid leaf growth and bulking rather than the later period of bulking.

## Modern breeding strategies for drought tolerance

Current objectives for breeding for drought tolerance in several cassava breeding programmes include: (1) characterization of germplasm for tolerance to extended water shortages, either natural or imposed, and to low-fertility soils; (2) characterization of germplasm for vigor under early drought (within the first 3 months); (3) study of leaf photosynthetic potential in relation to productivity under various edaphoclimatic conditions; and (4) identification of other plant traits that might be of use in cassava improvement. Breeding substantially until recently has been based on classical approach.

Selection of parental materials for tolerance to water stress and infertile soils has resulted in breeding improved germplasm adapted to both stress environments. The International Fund for Agricultural Development (IFAD) has supported a long-term project for selection in different semi-arid environments in Northeastern Brazil—where the greatest genetic diversity of cassava germplasm for adaptation to drought is found—and distribution of the elite germplasm throughout Africa. Prior to screening for drought tolerance, it is important to incorporate CMD resistance for Africa as a whole, as well as cassava brown streak disease (CBSD) field resistance for Eastern and Southern Africa. Otherwise, the effects of these diseases can mask the plant's response to drought.

The immense diversity of environmental components in major drought-prone areas of the world poses difficulties in planning specific crosses and in selecting breeding materials that will suit the specific ecological conditions peculiar to a site. A systematic and uniform characterization of the pertinent environment factors for important drought-affected areas would guide researchers in formulating breeding objectives and procedures, and greatly accelerate the impact of breeding programmes. CIAT and other breeding programmes in Latin America and the Caribbean have used this systematic approach. Adopting appropriate selection criteria is very important. They should include rapid, inexpensive and simple methodologies, and should be based on physiological interaction of drought with crop growth and yield. Any criteria based on a variety's ability to maintain a high water status and efficient water use would clearly relate to productivity. By diagnosing environmental factors prevailing in different drought-prone areas, the breeder is in a better position to incorporate specific drought-resistance mechanisms and recovery capabilities into breeding populations.

In Nigeria which is the world largest producer of cassava, its national research center for cassava, the National Root Crops Research Institute (NRCRI) is massively screening elite germplasm to identify genotypic responses to drought that can selectively be hybridized and recombined to develop an array of genotypes adapted to the various needs of drought-prone areas. The selection of progenies at drought-prone sites is critical for the identification of genotypes that perform well under water stress.

In previous breeding activities at IITA, three factors were considered in relation to drought tolerance: (1) the timing and length of water stress; (2) yield under different water regimes, or at different sites; and (3) drought reactions scored for vegetative growth during the dry season. This form of multicriterion comparison has provided more meaningful interpretations of varietal differences in agronomic performance related to drought than comparison of absolute yields. Varieties with both drought resistance and good recovery ability are key requirements for stable performance in areas with a longer rainy season or those with a bimodal rainfall pattern that are also prone to occasional prolonged drought periods.

Given the vast array of opportunities of molecular resources being generated and available to cassava, molecular breeding is now rapidly evolving for the crop. From the initial molecular breeding initiatives supported by the GCP since 2003, many breeding programmes have developed capacity to deploy molecular tools. The BMGF in 2012 is supporting a Cornell University led consortium to use of genomic selection in Africa to fast-track cassava breeding and is in the process generating and using huge assembly of sequence data (http://www.nextgencassava.org/). These developments are rapidly changing the landscape of breeding in Africa and globally. These rapid changes generally require faster phenotyping protocols and efficient genomic selection tools to increase genetic gain and expedite product delivery of drought tolerance products. Some of the modern breeding strategies require minimal phenotyping or early and fast phenotyping protocols.

Breeding for complex traits is expensive due to the need for highly replicated phenotyping trials over several environments. This justifies the quest for a MAB approach that increases precision of selection and reduces the requirement for phenotyping. MARS is a MAB strategy for forward breeding of genes and QTLs for relatively complex traits (Ribaut and Betran, 1999; Ragot et al., 2000; Eathington, 2005; Crosbie et al., 2006) such a drought tolerance. It is a genotype construction process that increases the frequency of beneficial alleles and aids the development of genotypes with the best haplotype combination at selected loci in the genome. A typical MARS scheme is illustrated in Figure 2. Under the GCP—Cassava Challenge Initiative, African breeding programs have initiated MARS for drought tolerance breeding in cassava. SSR and SNP markers are used to identify QTLs and then to identify important allele combinations through three cycles of selection, which is only then which is only then followed by phenotyping.

Genomic Selection is an alternative approach well suited to complex traits (Meuwissen et al., 2001). This approach depends on high-throughput genotyping and novel statistical methods. GS uses all marker data as predictors of performance, thus enabling the selection for multiple loci of small genetic effect (Jannink et al., 2010). Essentially, breeding populations are extensively genotyped (using next generation sequencing technologies) to give full genome coverage and phenotyped to create models that calculate genomic estimates of breeding values (GEBVs) which are used to select candidate parents. These values can then be used for selection within a breeding population, without the need for phenotypic evaluation. This new breeding approach have strong significant benefits in breeding for drought tolerance and quantitative traits in highly heterozygous species as cassava (Heffner et al., 2009; Jannink et al., 2010).

Genome wide selection (GWS) is a strategy found suitable for complex traits controlled by many QTLs and with a low *h*^2^. GWS can be implemented in the same way as MARS except that all individuals are genotyped with a large number of markers (Ferguson et al., 2012b). Genome wide selection (Meuwissen et al., 2001) determines prediction of performance based on as many loci as possible (unlimited number) without QTL mapping. In GWS, trait values are predicted from a weighted index calculated for each marker. Simulation studies have indicated that across different numbers of QTL (20, 40, and 100) and levels of *h*^2^, responses to genome wide selection were 18–43% larger than the corresponding responses to MARS (Bernardo and Yu, 2007).

## Key early-growth phase phenotyping methodology for molecular breeding

A key focus for modern breeding is the need to rapidly make genetic advances and reduce the breeding scheme by efficiently stack traits using both molecular tools and efficient phenotyping strategies. This is more challenging for complex traits and especially for those that are often evaluated very late in the growth cycle. While a good number of traits often evaluated at harvest periods are strong drought tolerance determinants, their late measurements make them undesirable to meet the objectives of modern breeding that seeks a fast screening procedure and quick systematic elimination to reduce population sizes that are measured late in breeding scheme. Modified traits that have key predictive power to estimate yield and adaptation potential in drought prone environments and which can easily be assessed early in the growth cycle are target traits of interests for modern breeding. Recently three modified traits have proved very useful based on their recent application in drought phenotyping within a 7–8 month evaluation cycle in contrast to a 12–18 month cycle that typically applies under drought stress ecologies.

### Bulking at 7 map

Bulking in cassava refers to the swelling or thickening of the storage roots as they are filled with excess assimilates after the plant might have satisfied the needs for vegetative growth. Early bulking has been used as concept to described early maturing cassava or early-ready cassava varieties that are harvestable at 7–8 months. The food security role of cassava in averting famine has necessitated the need for early-ready varieties in contrast to late yielding varieties. However, early bulking has been a trait mainly evaluated mainly in humid agro-ecologies where conditions are rather optimal for growth.

In the dry ecology, drought imposes slow crop development that makes harvest of cassava to extend beyond 12 months and sometimes between 15 and 18 MAP. Early bulking is therefore seldom considered as a measurable trait in marginal environment. The need to use as an early screening procedure has rapidly become important given the need to accelerate phenotyping for assessing productivity in drought prone environments. Early bulking in dry ecologies is rather implied to identify good bulkers under stress rather than identifying early maturing varieties. Thus evaluating early bulking for drought tolerance is used to select good varieties potential good yield at 12 MAP. So it is a fast screening method to select for yield under drought tolerance *per se*. This have been applied in recent studies for drought adaptation.

**Figure 3:**
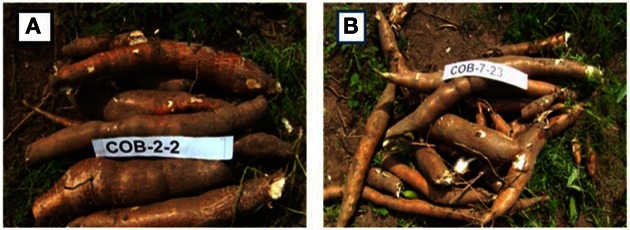
**Good bulking genotypes (at 7 months after planting) developed in the Cassava breeding programme at National Root Crops Research Institute, Umudike, Nigeria. (A)** Early bulking genotype with big sized commercial roots at 7 MAP. **(B)** Early bulking genotype with moderate sized commercial roots at 7 MAP.

A study of early bulking was conducted in the guinea savannah (Olasanmi, 2010). Dry season could vary from 4 to 6 months in the savanna zone requiring cassava varieties to have good adaptation for drought tolerance to enhance good productivity in these ecologies as well. Some pre-selected cassava genotypes with good bulking (Figure 3) were evaluated by NRCRI in 2010 and 2011 at Otobi (derived savanna) (Table 1). They were evaluated for early bulking in terms of root yield and other related parameters at 7 months after planting. The objective was to test the hypothesis that early bulkers could be used as a identify potentially good productive genotypes at 12 months in regions were drought stress could be a severe limitation to productivity.

**Table 1:** **Fresh root yield and other yield related attributes of early bulking cassava genotypes at two harvest dates at Otobi, Nigeria (source Olasanmi, 2010)**.

**Genotype**	**7 MAP**		**12 MAP**		**Yield increase**	**% Yield increase**
	**FRY (t/ha)**	**PI**	**FRY (t/ha)**	**HI**		
COB-4-52	2.33	0.16	10.51	0.41	8.18	350.6
COB-6-31	5.67	0.19	20.61	0.39	14.94	263.7
COB-5-86	9.96	0.32	35.86	0.40	25.90	260.2
COB-4-79	4.78	0.29	15.56	0.40	10.78	225.6
COB-1-139	9.31	0.34	30.11	0.45	20.81	223.6
COB-5-17	9.47	0.24	29.67	0.32	20.20	213.4
COB-7-180	7.59	0.19	22.41	0.30	14.82	195.3
COB-5-28	6.68	0.32	19.52	0.38	12.85	192.4
COB-5-44	9.78	0.22	28.33	0.24	18.56	189.8
COB-5-4	10.65	0.38	29.20	0.47	18.56	174.3
COB-4-100	10.56	0.29	27.66	0.37	17.11	162.0
COB-6-41	7.61	0.20	19.00	0.23	11.39	149.6
COB-7-197	13.93	0.30	33.85	0.36	19.92	143.0
COB-7-25	20.00	0.33	47.29	0.46	27.29	136.4
COB-5-53	6.35	0.37	14.31	0.45	7.96	125.5
COB-1-103	9.86	0.32	21.88	0.43	12.02	121.9
COB-4-75	13.68	0.36	26.81	0.44	13.13	96.0
COB-5-24	7.11	0.31	13.79	0.33	6.68	94.0
COB-4-77	11.13	0.33	21.59	0.43	10.46	93.9
COB-5-104	6.39	0.24	12.25	0.33	5.86	91.6
COB-4-74	8.79	0.28	16.78	0.42	7.99	91.0
COB-6-19	9.78	0.25	17.83	0.46	8.05	82.3
COB-5-12	10.65	0.29	18.87	0.34	8.22	77.2
COB-1-163	14.00	0.28	24.69	0.34	10.69	76.3
COB-5-57	11.25	0.25	19.77	0.28	8.52	75.8
COB-4-27	10.11	0.34	17.39	0.38	7.29	72.1
TMS 98/0505	11.24	0.21	18.51	0.27	7.26	64.6
COB-5-48	7.64	0.24	11.67	0.27	4.03	52.8
COB-5-11	11.46	0.28	17.46	0.36	6.00	52.4
COB-6-4	12.54	0.24	18.95	0.28	6.42	51.2
COB-5-61	12.07	0.32	16.20	0.33	4.13	34.3
TMS 30572	14.71	0.27	18.90	0.31	4.19	28.4
COB-5-36	9.25	0.24	11.45	0.22	2.20	23.8
COB-6-1	14.56	0.33	12.50	0.30	-2.06	-14.1

MAP, months after planting; FRY, fresh root yield; HI, harvest index; PI, partioning index.

In the study a set of 33 early bulking cassava genotypes and two check varieties (TMS 30572 and TMS 98/0505) were evaluated for yield at 7 and 12 MAP. A new parameter which was used to assess tolerance is the relative increase in yield from 7 MAP to 12 MAP. Fresh root yield and other yield related attributes of the early bulking cassava genotypes evaluated at Otobi are shown in Table 1. The average yield at 7 MAP was 10 t ha^−1^ at Otobi in the derived savanna while it 21.2 t ha^−1^ at Otobi. Results indicate that genotypes that were good bulking at 7 MAP relatively maintained good yields at 12 MAP. At Otobi, about 81% of the genotypes having about 20 t ha^−1^ or more at 12 MAP had 9–10 t ha^−1^ at 7 MAP. The results therefore shows that early bulking could be used as a useful parameter to screen for productivity at 12 MAP. Such genotypes are likely to maximize available moisture for bulking to improve yields. These materials are being planned for further test in semi-arid zones (Sudan and Sahel savannas). Yield differences between 7 MAP and 12 MAP tend to indicate that 18 (54.5%) of the genotypes based on the results were very good bulking materials with less than 55% root yield increase at 12 MAP over root yield at 7 MAP. Genotypes with good bulking at 7 MAP and without highly extended yield increase are considered more drought tolerant. The results obtained showed that 7-month bulking assessment in ecologies with high drought stress could be used as a good trait to rapidly screen for drought tolerance under modern breeding. Efforts to screen for bulking at 5 MAP for productivity potential at 12 MAP are underway.

### Partitioning index

Harvest index (HI) is the ratio of economic yield to that of biomass yield of a crop and is typically measured at 12 MAP. Molecular breeding essentially requires rapid screening methodology that necessitates quick prediction for good partioning to estimate yield potential. HI at 12 MAP is rather late in the growth cycle and makes it not readily ideal for early screening of breeding populations for drought adaptation in the cassava breeding scheme.

The ability to estimate quick partitioning of assimates at the early growth phase is therefore considered more desirable. Recent initiatives to assess partioning index at the early stages have been explored to improve rapid screening for good yield. Duque (2012) examined 45 diverse cassava genotypes representing a range of reported drought tolerances from among collections at CIAT and Embrapa. The studies were done on potted plants so that water supply in well-watered and water-stressed treatments could be controlled. Partioning index which is the ratio of the storage root weight as a fraction of the total plant biomass at 4–5 months was correlated with harvest index at 12 MAP. The correlation between storage root mass and the partitioning ratio of storage root biomass:total plant biomass was found high, especially underwater stress (Duque, 2012). The study showed that the best genotypes maintain a robust developmental programme that sustains storage root growth in the face of water stress, whereas poorer genotypes allow storage root growth to suffer at the expense of other growing plant organs. In terms of phenotyping strategies, the study suggests that evaluation of biomass partitioning ratio at an early stage of storage root development could be a useful indicator of a genotype's tendency to favor storage root growth when resources are limited by water stress.

Findings in a study reported by Olasanmi (2010) has also shown good correlation between partioning index at (7 MAP) and harvest index (12 MAP) for drought tolerant genotypes and might be useful as a critical screening method for preliminary selection for drought adaptation evaluation. Results obtained in the study (at Umudike—humid ecology; and Otobi—Guinea savanna) indicated that genotypes that had a PI of 0.3 tended to produce better and maintained good HI at 12 MAP. In the study, the difference between PI and HI among three classes of cassava (early bulkers, medium bulker and late bulkers) at two harvesting age (7 and 12 months after planting) were significant. The difference was widest for the late bulkers than the other two classes (Table 2). Medium cassava bulkers are those that are intermediate between the early and late types. Due to late bulking, the PI does not necessarily correlate with HI at 12 MAP for this group. PI therefore tend to predict stronger for early bulking genotypes as shown by the results (Olasanmi, 2010), The implication for late bulkers in dry ecologies is that due to drought effect, it may likely attain maturity very late often well beyond 12 MAP for good and reasonable yield to be attained. The use of PI could rapidly allow breeders to cut down on the population and thus accelerate rapid selection thus reducing the breeding scheme through shortened phenotyping regimes.

**Table 2:** **Average harvest index (HI) among different bulking rate groups of cassava at two locations in Nigeria**.

**Location**	**Late bulkers**		**Medium bulkers**		**Early bulkers**	
	**7 MAP**	**12 MAP**	**7 MAP**	**12 MAP**	**7 MAP**	**12 MAP**
Umudike	0.32	0.59	0.42	0.59	0.45	0.57
Otobi	0.28	0.38	0.28	0.35	0.29	0.29
	**Difference between PI and HI between the two harvesting age**					
	**Late bulkers**		**Medium bulkers**		**Early bulkers**	
Umudike	0.27		0.17		0.12	
Otobi	0.10		0.07		0.00	

### Stem starch content

Cassava is vegetatively propagated. The size and quality of stem are of fundamental importance for high yields (Eke-Okoro et al., 2001). Differences in weight of stem cuttings result in differences in food reserve (Okeke, 1998), and it is on this that the initial growth of the plant depends implying basically that stem weight or starch are associated to the establishment phase of cassava in the field. This has recently been explored for drought tolerance phenotyping. The establishment phase of cassava is critical to rapid adaptation of cassava and has been hypothesized as more critical under water stress either at the initiation of the growth phase or during prolonged stress when food reserves are mobilized to sustain metabolic activities.

**Figure 4:**
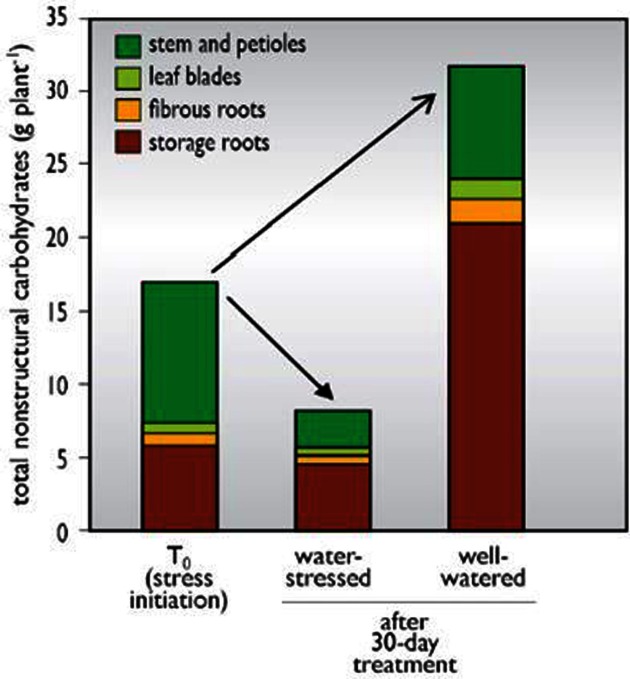
**Accumulation of total non-structural carbohydrates in cassava plant parts during initial growth and during a 40 subsequent period of water-stressed or well-watered conditions.** Source: (Duque, 2012).

**Figure 5:**
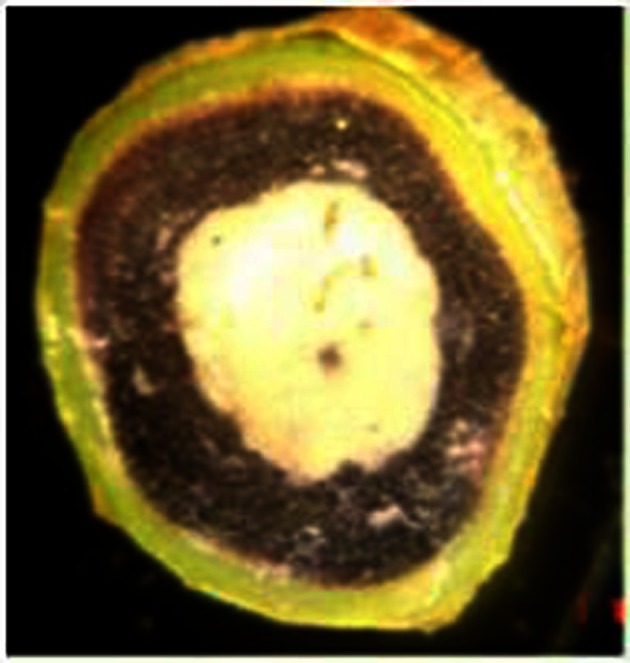
**Starch in cassava stems—remobilized during stress (staining with iodine).** Source: Duque, 2012.

Given that water stress diminishes photosynthetic carbon fixation, and yet cassava can retain the ability to resume growth after long drought periods, it has been hypothesized that carbohydrate storage reserves in cassava's thick robust stems might provide a supply of carbohydrate to sustain meristems and other respiring organs during prolonged stress. (Duque, 2012) studied found that reserves in leaf blades were limited and these reserves were depleted rapidly during stress (Figure 4). In contrast, stems and storage roots maintained a relatively high starch content per organ from treatment initiation to the final harvest. Total non-structural carbohydrate (TNC) content per plant was maintained in storage roots through the entirety of the experiment, while the stem became a source of slowly remobilized starch during stress. The amount of starch stored in stems was considerable, representing about 35% of the TNC in the plant at stress initiation (T_0_), and 6% of total plant dry mass. These data suggest that this pool of TNC reserves is important in sustaining meristems and other respiring organs during prolonged stress. Duque (2012) showed that cassava stems accumulate starch (Figure 5) gradually over a 45-day period of growth after seedling establishment, in advance of storage root bulking (Table 3). In studies with 15 diverse genotypes, fresh root biomass production under stress correlated with the extent to which a genotype accumulated starch in its stems. Collectively, these studies suggest that the extent to which stems accumulate starch in advance of water stress could be a valuable trait for drought tolerance.

**Table 3:** **Carbohydrate accumulation in the initial growth period after seedling establishment of cassava in the Corpoica (Corporación Colombiana de Investigación Agropecuaria; Colombian Corporation for Agricultural Research) field sites at Turipana and El Guamo in Colombia. An average of 15 genotypes is shown (source: Duque, 2012)**.

	**Carbohydrate (μmol g^−1^ dry weight)**			
	**Leaf blade**		**Stems**	
**Days from plant establishment**	**Total sugars**	**Starch**	**Total sugars**	**Starch**
0	75	15	121	293
15	78	14	178	332
30	79	19	192	659
45	101	5	239	1016

## Future direction: phenomics and high throughput phenotying

Phenomics is the systematic study of phenotypes on a genome-wide scale and defines phenotypic features across multiple levels of expression. It involves a large-scale phenotypic data collection and analysis, and thus enables the characterization of phenotypes in a rigorous and efficient way, to link traits with the associated genes. Phenomics is normally conducted by running multiple phenotypic assays on a large set of genotypes. Phenotypic parameters cover morphological measures (plant height), dynamic measures (metabolism) and molecular measures (transcript profiles). Developing strong capacity for cassava phenomics is essential to high throughput phenotyping to close the gap between plant physiology and genetics (Furbank, 2009). High-throughput phenotyping technologies will be particularly important for studies of drought tolerance.

High throughput phenotyping for target and correlated traits is increasingly becoming important in crops. However, there are still many important traits that are difficult to evaluate. Phenomics technologies could bring new approaches to address these challenges to efficiently identify superior genotypes and train prediction models. Improved methods are required needed for high-throughput collection of diverse phenotypic measures, in the field and controlled environments. Some of the high throughput technologies used in drought studies in several crops include imaging systems, remote sensing, canopy spectral reflectance (for water use efficiency), etc. Cassava can immensely benefit from the use of such technologies.

Information about the physiological changes in response to drought over time is vital to identification and characterization of the different drought-tolerance mechanisms. This has been demonstrated in many crops based on high throughput technologies. For example, Image-based phenotyping offers a way to capture and extract morphological and developmental phenotype data, through non-destructive close-range or remote-sensing technologies. Remote sensing, an increasingly powerful tool has long been used in an attempt to measure the water status of individual plants or canopies (Blum et al., 1982). The most frequently used technique is thermal infrared imaging, or infrared thermography (IRT), to measure the leaf or canopy temperature which is drought parameter related to the extent of stomatal opening and evaporative cooling often measured (Balota et al., 2008). The use of thermal cameras for canopy temperature measurement offer a key benefit compared with temperature sensors (thermometers) as a facility for spatial resolution. It thus allows more precise measurements in a fraction of the time needed to perform several replicate readings per plot than an infrared thermometer, which is prone to error due to changing environmental conditions between measurements. In addition, a large number of plots in a field trial can be imaged at the same time, ideally allowing a comparison of differences in canopy temperature among genotypes without the need for normalization to determine the absolute leaf temperature (Jones et al., 2009).

Non-destructive imaging techniques allow a temporal resolution and monitoring of the same plants throughout the experiment. The development of good image systems that avoid destructive sampling will be very critical for root and tuber crops like cassava when storage root development is critical for assessment of drought on growth and development in the crop's growth cycle. The Combination of high throughput technologies have the huge potential to increase the power of data analysis. For example the combination of color and thermal imaging, has been indicated to increase the information and precision of leaf temperature measurements compared with thermal imaging alone (Berger et al., 2010). Although cassava have yet to significantly deploy high throughput technologies in drought studies, it is expected that this will change as cassava phenomics improve.

Despite the array of data characterizing water deficit responses that may relate to dehydration tolerance, there is still little understanding as to which responses, whether at the gene or cellular level, are actually adaptive in nature and truly critical for or central to tolerance (Bray, 2002). Metabolite profiling offers strong opportunities to remedy these gaps. Some of the most important responses of a plant against drought stress are associated with the accumulation of minerals (Samarah et al., 2004) and the enhanced synthesis of osmoprotectants, osmolytes, anti oxidants, or compatible solutes, which are part of normal metabolism. The accumulation of these compounds helps the stressed cells in water retention (Hare et al., 1998; Setter, 2012) and in the maintenance of the structural integrity of the cell membranes (Conroy et al., 1988). Metabolic profiles have the potential to uncover a cascade of biochemical regulation strategies that may be explored to enhance drought tolerance in crops (Setter, 2012). Mass spectrometry (MS) and Nuclear magnetic resonance (NMR) spectroscopy are used to identify and to quantify metabolites. Nuclear magnetic resonance (NMR) spectroscopy can be used to monitor and quantify the degree of metabolic impact induced by drought or other environmental disturbances (Bligny and Douce, 2001; Charlton et al., 2008), since NMR can bring “high-throughput” spectroscopic/structural information on a wide range of metabolites simultaneously with high analytical precision.

Phenomic datasets can be large and complex and appropriate management systems are required to enhance analysis. The power of phenomics is largely expected to be enhanced when datasets are combined and correlated across different studies. Considering the complexity of both drought and plant responses to drought, trait dissection effected by high-throughput phenotyping provides strong process to understand plant responses to drought, and its genetic basis for effective application to improve crop performance and yield under a variety of drought conditions in crops (Berger et al., 2010).

In cassava, drought phenotyping has with morpho-physiological and agronomic traits that does not integratively provide sufficient understanding to drought tolerance in this crop. The increasing genomic resources arising from the use of next generation sequencing technologies and GBS in cassava implies that the quantum of genotypic data or information being generated can only be meaningfully analyzed and applied by improving capacity in phenomics. High throughput phenotyping will be required to complement molecular tools for rapid genetic gain for drought tolerance in a fast track breeding scheme.

## Conclusions

A review of the literature on drought tolerance in cassava reveals the physiological basis of drought tolerance in cassava and its integration with agronomic traits. Some traits are not easily phenotyped. For example, a deeper root system provides access to more soil water for the crop during drought. Basically, breeding and selection based on root system evaluation have not been well explored, and simple methods to evaluate root systems have yet to be developed in cassava.

A good number of the conventional traits typically used to assess drought adaptation, though relevant have limitations especially for those that are measured late in the growth cycle which makes current efforts to reduce the breeding cycle a challenge. The use of molecular tools in breeding is designed to efficiently select for genes for rapid genetic advances in the breeding (especially for complex traits). The strength and beneficial aspect of molecular tools lies in fast tracking the development of varieties that maximize gene combinations for complex traits. Molecular breeding thus require strategies that not only support fast rapid phenotyping protocols but minimal phenotyping. In such scenario, many of the current traits are not well suited to the modern breeding paradigms. Therefore the need to identify more efficient and rapid and or simple phenotyping protocols are expected to increase. Drought adaptation traits that may easily be used to assess productivity at early growth phase may be a quick strategy to accelerate drought tolerance selection in modern breeding for cassava. Physiological traits such as stomatal conductance and leaf photosynthesis that are easily measured will continue to be favored traits in modern breeding.

Complementary phenotyping strategies such as metabolite profiling used in combination with conventional cassava drought phenotyping traits will further enhance our understanding of drought tolerance in cassava. While rapid advances in high throughput genotyping has been achieved, much have yet to be done for phenotyping for cassava. The power to detect useful genes and understand the metabolic pathways of drought tolerance can only be efficiently dissected by complementing it with high through phenotyping that enhances quality of phenotypic data both in precision and accuracy. Developing strong phenomic capacity for drought tolerance in cassava is therefore crucial to the process. Significant progress in drought tolerance breeding will largely depend on how quickly this capacity is developed.

### Conflict of interest statement

The authors declare that the research was conducted in the absence of any commercial or financial relationships that could be construed as a potential conflict of interest.

## Footnotes

^1^http://gcpcr.grinfo.net/index.php?offset=0&app=datasets&inc=search_simple&gcpcr_search_string=Cassava&orderby=&orderdesc=false&limit=10#search_results

^2^At Clemson University, Clemson, South Carolina, USA; http://www.genome.clemson.edu/

^3^http://www.454.com/

^4^www.phytozome.net/cassava
